# Variation of Structure and Photoluminescence Properties of Ce^3+^ Doped MgAlON Transparent Ceramics with Different Doping Content

**DOI:** 10.3390/ma10070792

**Published:** 2017-07-13

**Authors:** Xin Liu, Bowen Chen, Bingtian Tu, Hao Wang, Weimin Wang, Zhengyi Fu

**Affiliations:** State Key Lab of Advanced Technology for Materials Synthesis and Processing, Wuhan University of Technology, Wuhan 430070, China; liuxinshs@gmail.com (X.L.); chenbowen1002@sohu.com (B.C.); tubt@whut.edu.cn (B.T.); shswmwang@whut.edu.cn (W.W.); zyfu@whut.edu.cn (Z.F.)

**Keywords:** fluorescent transparent ceramics, MgAlON:Ce, structure, cathodoluminescence, photoluminescence

## Abstract

Transparent MgAlON:Ce fluorescent ceramics with doping content of 0.005, 0.01, and 0.02 at % were fabricated by pressureless sintering. All the samples were dense and large in grain size. Under the excitation of 320 nm UV, the samples emitted blue lights centered around 410 nm. The 0.005 and 0.01 at % Ce^3+^ doped samples were single phase by XRD detection, and possessed good optical and mechanical properties, and luminous thermal stability. The fluorescence lifetime, the CL and PL spectra analysis, indicated that most of the luminous centers were segregated in GB, and there was still a small part of second phase existing in 0.01 at % Ce^3+^ doped sample, which revealed that spectroscopy methods possessed better sensitivity in smaller scale and lower concentration detection than XRD. As the doping content increasing to 0.02 at %, a mass of second phase arose, which resulted in the optical transparency, mechanical property, luminous thermal stability decline, and the PL spectra red shift by the formation of second phase. It revealed that the performances were fatally deteriorated by excess doping of Ce^3+^ ions.

## 1. Introduction

As a special kind of inorganic-nonmetal materials with excellent physicochemical properties, transparent ceramics have drawn much attention since the fabrication of first transparent alumina by General Electric [1,2]. In recent years, functionalizing transparent ceramics by doping activator ions to gain brand new properties and broad application fields has become a hot topic in research. Thanks to the diverse optical energy transition abilities of the rare earth activator ions, the functionalized transparent ceramics could be potentially used in high power LED devices [3,4], high performance lasers and scintillators [5,6], UV wiping-off windows [7], and so on.

For the above mentioned optical transparent energy transfer materials, two major impacts which seriously affect the performance should be considered. One is the optical transparency. Relatively high transparency always means large flux of incident and transmission light, which is in favor of the energy usage ratio to the materials [8]. Another factor is the optical energy conversion efficiency of the appropriate volume activator ions. In other words, an appropriate doping concentration with proper luminescence centers where no second phase and concentration quenching are necessary. For a specific activator, local structure defects—like the surrounding impurities and crystal dislocation areas—are the main source of extra energy dissipation [9]. Therefore, to ensure the materials perform efficiently, the transparency and optical absorption-conversion efficiency should be considered. As, in most cases, both of the above-mentioned factors jointly affect the performance.

In our earlier work, the highly transparent cubic spinel type MgAlON transparent ceramic, whose space group is Fd-3m, was firstly fabricated [10] and a few relatively optical functionalization works had been completed, such as MgAlON:Mn [11], MgAlON:Eu [7], MgAlON:Ce [12]. On account of the small volume of polyhedra in MgAlON crystal structure, the large-size rare earth ions could not dissolve in the crystal lattice in quantity [13]. Though the upper materials possessed good transparency and photoluminescence thermal stability, the doping concentration was signally inferior to commonly used YAG:Ce [14], which could lead to energy transfer inefficiency by the minor luminescence centers. To improve the aforementioned problem, a larger doping concentration of Ce^3+^ could be considered. While, several problems should be taken into account by increasing the Ce^3+^ ions: (1) acting as sintering aids, the increasing of rare earth oxides could affect the microstructure of the ceramics [15,16]. Afterwards, the optical transparency and mechanical properties should be influenced, as well as the energy conversion efficiency; (2) more serious segregation of Ce^3+^ in grain boundaries (GB) could generate the negative effects, such as the interatomic energy transfer consumption, variation of crystalline field, and formation of second phase.

To come into a proper concentration of Ce^3+^ in MgAlON transparent ceramic hosts, understanding the effects of composition and structure, optical and mechanical properties, as well as photoluminescence behaviors with Ce^3+^ content increasing, in this work, the phase composition, microstructure and the closely related optical transparency and mechanical properties of various Ce^3+^ doped MgAlON fluorescence transparent ceramics were investigated. The light excitation and emission properties introduced by the addition of rare earth cations were characterized. The GB segregation behavior of rare earth ions was studied. The relationship of phase composition, local environment of rare earth ions, and the optical transparency and photoluminescence properties of the samples were finally established.

## 2. Experimental Procedures

In this work, fine MgAlON ceramic powders, whose chemical formula is Mg_0.27_Al_2.58_O_3.73_N_0.27_, were prepared by solid-state reaction process previously. The as-prepared MgAlON powders were mixed with different content of CeO_2_ (>99.99%, Alfa Aesar (Shanghai, China)) by ball-milling with alcohol media and four times weight of Al_2_O_3_ balls for 12 h. The addition contents of CeO_2_ were based on the Ce/Al ratios, which were 0.005, 0.01, 0.02 at % (atom ratios) respectively. Then these sizing agents were dried and sieved. The pretreated powders were firstly dry-pressed to a disk of 20 mm in diameter and 4 mm in thickness by a uniaxial pressure of 20 MPa, then treated by cold isostatic pressing (CIP) at 200 MPa. The obtained green bodies were sintered at 1875 °C for 24 h in N_2_ by a graphite furnace, and then freely cooled to room temperature. The ceramics were ground to 2 mm thickness and mirror-polished finally.

The phase composition of obtained transparent ceramics were obtained by the X-ray diffractometry (XRD; X’pert PRO of Panalytical, Almelo, The Netherlands) with stepping rate of 2°/min from 15° to 85°. The scanning electron microscope (SEM; S-3400, Hitachi, Tokyo, Japan) was employed to characterize the microstructure of ceramics surface with acceleration voltage of 15 KV, the equipped energy disperse spectroscopy (EDS; EDX, Oxford Instruments, Oxford, UK) was applied to identify elements composition. The Vickers hardness was measured using the hardness tester (VHT; Model 430 SVD, Wolpert, Shanghai, China) at a load of 49 N for 10 s. The ultraviolet-visible spectrophotometer (UV–Vis; UV-2550, Shimadzu, Kyoto, Japan) was used to measure the transmittance of samples ranging from 200 to 800 nm with stepping of 1 nm. The photoluminescence spectra at room temperature (RT), decay times, quantum efficiency (QE) and temperature-dependent emission spectra from 323 to 523 K were recorded by a fluorophotometer (FP; FLS920, Edinburgh Instruments, Livingston, UK) with stepping of 0.1 nm. The excitation and emission light of transparent ceramics were measured by a BaSO_4_-coated integrating sphere attached to the spectrofluorometer using a calibrated tungsten lamp and the internal QEs were calculated by the following equation [17]

(1) $$\eta =\frac{L_{sample}}{E_{reference}-E_{sample}}$$

where *η* represented the internal QE, *L_sample_* was the emission intensity of samples, *E_reference_* and *E_sample_* were the intensities of the excitation light not absorbed by the undoped MgAlON transparent ceramic (reference) and the Ce^3+^ doped samples, respectively. The CL mapping images and spectra of the unetched MgAlON:Ce ceramic surface were obtained by a field-emission gun scanning electron microscope (FESEM; Quanta 200 F, FEI, Hillsboro, OR, USA) combined with a CL device (CL; Mono Cl^3+^, Gatan, Pleasanton, CA, USA) with 15 KV acceleration voltage and 5.0 spot size. Density measurements of the samples were carried out in deionized water at 26 °C by Archimedes method through a densitometer (DM; BT 124 S, Sartorius, Göttingen, Germany).

## 3. Results and Discussion

### 3.1. Phase and Microstructure

**Figure 1:**
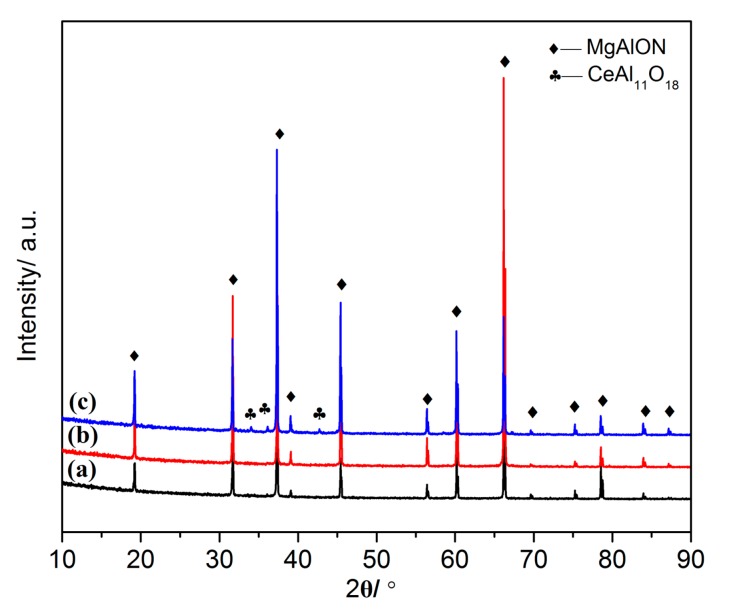
XRD patterns of MgAlON: Ce^3+^ fluorescent transparent ceramics with doping contents as: (**a**) 0.005; (**b**) 0.01; (**c**) 0.02 at %.

The XRD patterns of MgAlON:Ce fluorescence transparent ceramics with Ce^3+^ content of 0.005, 0.01, 0.02 at % were shown in Figure 1, respectively. The 0.005 and 0.01 at % doping content MgAlON:Ce ceramics were only consist of MgAlON phase, while the extra second phase identified as CeAl_11_O_18_ type was found when the doping content reached to 0.02 at %. The phase composition analysis of samples indicated that MgAlON transparent ceramic host could not accommodate the Ce^3+^ ions plentifully. In our previous work, the MgAlON:Eu transparent ceramic remained single phase when the doping content of Eu^2+^ was as high as 0.15 at %. There are no apparent peak shifts of MgAlON phase in the patterns, which implied that no cell parameters change the MgAlON host with the increase of Ce^3+^, according to the Bragg diffraction equation. The main reason should be owing to the fact that almost all of the Ce^3+^ ions were segregated in the GB of the MgAlON ceramic.

**Figure 2:**
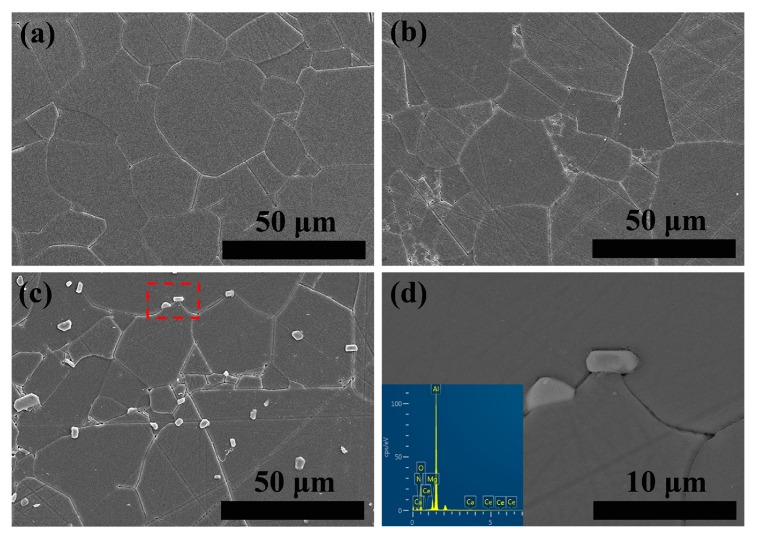
SEM images of MgAlON: Ce^3+^ fluorescent transparent ceramics with (**a**) 0.005; (**b**) 0.01; (**c**) 0.02 at % doping contents and (**d**) BSE image and EDS element analysis of second phase.

The microstructure of the chemically etched MgAlON:Ce fluorescence transparent ceramics were demonstrated in Figure 2. The surfaces of 0.005 and 0.01 at % Ce^3+^ doped samples separately shown in Figure 2a,b appeared smooth and dense with grain sizes centered in 20~50 μm, while a few particles with several micron size adhered to the surface of the 0.02 at % Ce^3+^ doped sample were shown in Figure 2c. Through the brightness contrast in the BSE image, the Figure 2d, one could clearly make out that the particles were second phase. The EDS analysis result revealed that the particles contained Ca and Ce elements, which indicated that a portion of Ce^3+^ in CeAl_11_O_18_ crystal structure were taken place by Ca^2+^ that introduced from the raw materials. There were noticeably large differences among grain sizes (35 ± 17.8 μm) in the 0.02 at % Ce^3+^ doped sample, also regarded as abnormal grain growth, which should be caused by the relatively large amount of liquid phase produced in the sintering process.

### 3.2. Optical Transparency and Mechanical Property

**Figure 3:**
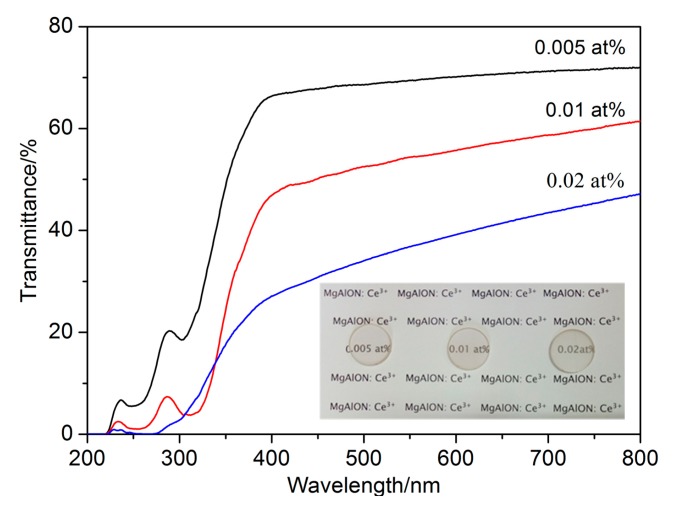
Optical transmittance in UV–Vis region of MgAlON: Ce^3+^ fluorescent transparent ceramics with various doping contents in 2 mm thickness and the photographs (insert).

As we know, the direction of light beam was changed and the in-line transmittance decreased when the incident light got through inhomogeneous medium with inconsistent refractive index [18]. In transparent ceramic bodies, all of the residual pores, second phases, or any other defects could act as optical loss initiators. The UV–Vis in-line transmittance spectra and photographs of the well-polished MgAlON:Ce samples were given in Figure 3. The 0.005 at % MgAlON:Ce ceramic possessed the highest in-line transmittance at about 68% at 400 nm and 72% at 800 nm, respectively, while it was still lower than the un-doped MgAlON. The relative density, which was the specific value of tested and theoretical density (3.646 g/cm^3^) shown in Table 1, was 99.5 ± 0.2% that meant a plenty of residual pores still existed. Considering the distribution behavior of Ce^3+^, the light scattering by pores and the GB segregated Ce^3+^ ions should be the main reasons for the reduction of in-line transmittance. The 0.01 at % Ce^3+^ doped MgAlON:Ce possessed relatively lower in-line transmittance due to the larger volume fraction of pores, which could be manifested in relative density (99.2 ± 0.5%) shown in Table 1. The in-line transmittance of 0.02 at % Ce^3+^ doped MgAlON:Ce samples decreased severely, which should be owing to the grievous light scattering loss caused by the vast of pores and second phase particles. By Ce^3+^ ions doping, extra optical energy absorption and transition mechanisms were implanted. Thus, remarkable transmittance declines occurred in the UV regions of all the samples.

The Vickers hardness of all the samples were provided in Table 1. With the increasing of doping contents, the Vickers hardness did not change evidently, the average value was about 13.9 GPa. That is, for the dense and large-grain transparent ceramics, the GB volume was limited. In other words, the mechanical properties could not be affected by the GB largely, but almost only be determined by grain properties.

### 3.3. Cathodoluminescence Mapping and Spectra

**Figure 4:**
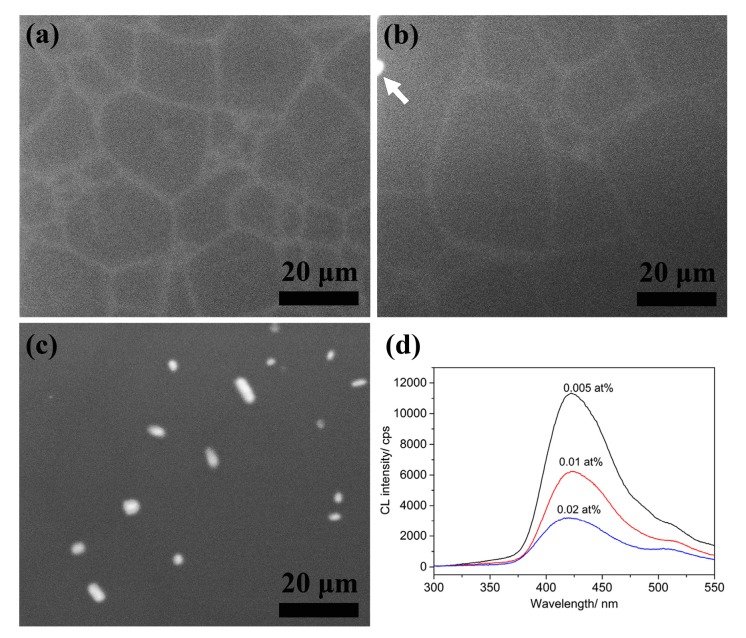
Luminescence centers mapping of MgAlON: Ce^3+^ fluorescent transparent ceramics with doping contents as (**a**) 0.005; (**b**) 0.01; (**c**) 0.02 at %; and (**d**) the CL spectrum.

Cathodoluminescence (CL) was a good method to analyze the properties of luminescence centers [19]. Figure 4 showed the CL mapping and spectra of the unetched MgAlON:Ce fluorescence transparent ceramic samples in various doping contents. The 0.005 at % Ce^3+^ doped MgAlON:Ce sample exhibited bright and conterminal lines on the surface in Figure 4a, which could be corresponded to the GB morphology shown in Figure 2. Therefore, the luminescence centers in samples with low doping contents should be concentrated on the GB region as previously reported. The mapping image of 0.02 at % Ce^3+^ doped MgAlON:Ce sample in Figure 4c revealed that just bright particles existed instead of conterminal lines. The luminescence centers from CeAl_11_O_18_ type second phase particles were relatively concentrated, compared to the GB distributed Ce^3+^ ions. Thus, the luminous of second phase particles covered one of the Ce^3+^ ion in GB. Sample with 0.01 at % content shown in Figure 4b consisted of blurry lines as well as few bright particles, which indicated that a small amount of second phase content formed. It illuminated that the maximum doping content of Ce^3+^ in MgAlON ceramic hosts was no more than 0.01 at %, and the spectroscopy possessed better sensitiveness to detect luminescent substances than XRD, which was proper in smaller scale and lower concentration.

The CL spectra of the samples were displayed in Figure 4d. The main emission peaks of samples were located at 420 nm, possibly owing to the Ce^3+^ emission in GB. With the increasing of doping contents, the proportion of peaks at about 510 nm increased. In view of the broad emission spectrum of CeAl_11_O_18_ ranging from 320 to 600 nm, the long wavelength light emission in CL spectra should be provided by the Ce^3+^ in CeAl_11_O_18_ type crystal structure.

### 3.4. Fluorescent Lifetime and Luminescent Sites

**Figure 5:**
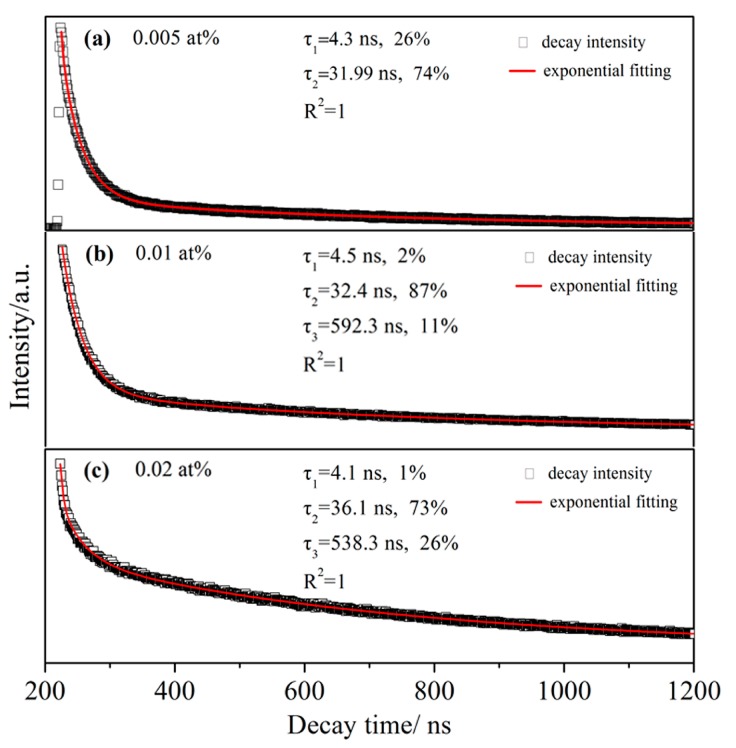
Decay time and fitting curves of MgAlON: Ce^3+^ fluorescent transparent ceramics with doping contents as (**a**) 0.005; (**b**) 0.01; (**c**) 0.02 at %.

Generally, the local structure dependent crystal field could severely affect the photoluminescence properties of the activator ions [20], especially for the 4f-5d energy transfer type rare earth ions, such as Ce^3+^ and Eu^2+^. As mentioned before, the large size Ce^3+^ ions were mainly concentrated in GB region. Presumably, a part of Ce^3+^ existed in MgAlON lattices near the GB, while remaining ones stayed in the expanding region of GB. The fluorescent lifetime was closely related to the charge transfer behavior. By fitting the lifetime, one could recognize the difference in local structures of the activator ions. To identify the change of luminescent sites by the increasing of the doping contents, fluorescent lifetimes and fitted curves of the samples were obtained in Figure 5. The fluorescent lifetime of 0.005 at % Ce^3+^ doped MgAlON:Ce sample illustrated in Figure 5a could be double exponential fitted by the following Equation (2)

(2) $$I(t)=I_{0}+\alpha \cdot e^{-t/\tau_{1}}+\beta \cdot e^{-t/\tau_{2}}$$

where *τ*_1_ and *τ*_2_ was the decay time, *α* and *β* were the contribution value of *τ*_1_ and *τ*_2_, respectively. The longer *τ*_2_ was 31.99 ns, which took 74% parts of the total luminescence centers. The remaining shorter *τ*_1_ was 4.3 ns. The luminescence centers in GB were supposed to have longer fluorescent lifetime due to the complex factors, such as the defect generated energy transfer. While luminescence centers in MgAlON lattices possessed single and fixed local structure, which contributed to the shorter parts of fluorescent lifetimes. As the doping contents of Ce^3+^ increased, the CL spectra reflected second phase appeared. The fluorescent lifetime of 0.01 and 0.02 at % Ce^3+^ doped MgAlON fluorescent transparent ceramics should be fitted by the multiple-exponential equation.

(3) $$I(t)=I_{0}+\alpha \cdot e^{-t/\tau_{1}}+\beta \cdot e^{-t/\tau_{2}}+\gamma \cdot e^{-t/\tau_{3}}$$

The fluorescent lifetime of 0.01at % Ce^3+^ doped MgAlON fluorescent transparent ceramic could be divided into three species, the shortest *τ*_1_ was 4.5 ns, longer *τ*_2_ was 32.4 ns, and the longest *τ*_3_ was 592.3 ns, with the portion as 2%, 87%, 11%, respectively. The CeAl_11_O_18_ type second phase contributed the longest part of fluorescent lifetime. When Ce^3+^ content increased to 0.02 at %,the decay time *τ*_1_, *τ*_2_, and *τ*_3_ slightly changed to 4.1, 36.1, 838.3 ns, while the portions were 1%, 73%, 26%. The variation of the portions revealed that a growing number of luminescence centers tended to segregate in GB as well as form second phase.

### 3.5. Photoluminescence (PL) Properties

**Figure 6:**
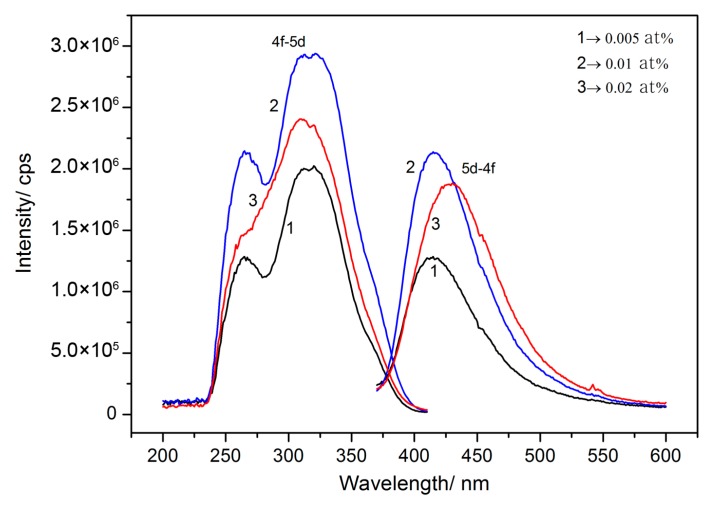
Excitation (monitored at 410 nm) and emission (excited at 320 nm) of MgAlON: Ce^3+^ fluorescent transparent ceramics with various doping contents at room temperature.

In order to illuminate the PL properties introduced by implanting of Ce^3+^ ions, the excitation and emission spectra of various doping contents of MgAlON:Ce were given in Figure 6. The emission spectra of the samples were generated by the 5d-4f energy transfer of Ce^3+^ ions, centered at about 410 nm, which were obviously distinct from Ce:YAG transparent ceramics (~520 nm) for the difference of host materials. Compared to the emission spectra of 0.005 and 0.01 at % samples, the intensities increased two-fold as the correspondent increasing ratio of doping content. On the basis of Beer-Lambert law, the incident *I*_0_ and transmission *I* intensity followed the Equation (4).

(4) $$I=I_{0}\cdot 10^{-Ecl}$$

where *E* was the constant, *c* stood for the concentration of the luminescent ionic, *l* was the thickness of the transparent medium. The luminescent intensity *F* was proportional to the intensity of absorption light (*I*_0_ − *I*), then the relationship was described as Equation (5).

(5) $$F=K^{\prime }(I_{0}-I)$$

where *K*′ was the constant. By combining Equations (4) and (5), when the *c* was pretty low, the *F* would be simplified as

(6) $$F=K^{\prime }I_{0}(1-e^{-2.3Ecl})=2.3K^{\prime }I_{0}Ecl=Kc$$

Here, the *K* was a new constant. As mentioned before, the luminescent intensity was determined by both transparency and optical absorption-conservation efficiency. While, the 0.005 at % Ce^3+^ doped sample was higher in transmittance than the 0.01 at % sample, the correspondent relationship between the intensities and doping contents revealed that the 0.01 at % sample should possess higher internal quantum efficiency (IQE).

Due to the severe decline of transmittance, the PL intensity of 0.02 at % Ce^3+^ doped sample decreased with the increase of the doping content. The IQE was 34%, which was closed to the 0.005 at % sample, given in Table 1. The IQE of 0.01 at % Ce^3+^ doped sample was 42%, which demonstrated the upper deduction. In the 0.02 at % Ce^3+^ doped sample, CeAl_11_O_18_ type second phase particles contributed parts of the long wavelength light emission [21], which increased the red-shift of the emission spectrum. The 4f-5d energy transfer absorption of Ce^3+^ ions (absorption peak at ~320 nm) and defects absorption (peak at ~260 nm) composed the excitation spectra of the Ce^3+^ doped MgAlON fluorescent transparent ceramics. Two characteristic peaks in the excitation spectrum of 0.02 at % sample overlapped, which revealed that the second phase also possessed strong absorption in the entire region of excitation spectra.

**Figure 7:**
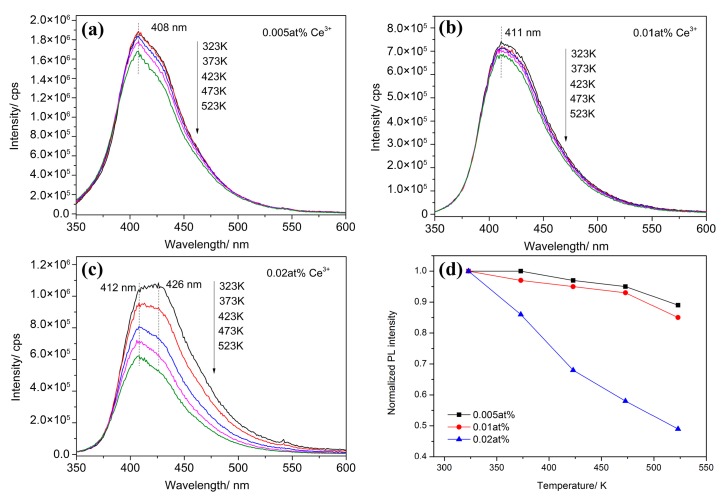
Temperature dependent emission spectra of (**a**) 0.005; (**b**) 0.01; (**c**) 0.02 at % Ce^3+^ doped MgAlON fluorescent transparent ceramics; and (**d**) the normalized photoluminescence intensity excited by 320 nm light.

As mentioned before, the importation of impurities could severely decreased the performance of fluorescent materials, especially the thermostability. To make the negative effects of excessive doping in MgAlON:Ce samples clear, the PL spectra at temperatures from 323 to 523 K were recorded and analyzed in Figure 7. The PL intensities all decreased as the increasing of temperature, which should be ascribed to the thermal activated phonon assisted excitation of Ce^3+^ ions, it was also called the thermal quenching [22]. From the normalized PL intensity given in Figure 7d, one can find that the PL intensity of 0.02 at % decreased a lot, only 48% of which from 323 K remained at 523 K. That means amount of defects arose at the same time of forming second phase, and they always promoted the nonradiative relaxation of the excitation electron. While, the 0.005 and 0.01 at % samples kept 90 and 85% of the 323 K intensity at 523 K, respectively. The temperature dependent emission spectra comparison of various Ce^3+^ doped MgAlON samples revealed that the second phase affected the fluorescent thermostability seriously, as well as shifted the PL spectra.

## 4. Conclusions

In this work, three doping contents (0.005, 0.01, and 0.02 at %) of MgAlON:Ce fluorescent transparent ceramics were fabricated by pressureless sintering. The 0.005 and 0.01 at % Ce^3+^ doped samples were single phase by XRD detection, and possessed good optical and mechanical properties. The transmittance of 0.005 at % sample was 68% at 400 nm and 72% at 800 nm. The Vickers hardness was about 13.8 GPa, IQE was 35 and 42%, respectively. While the fluorescence lifetime, CL and PL spectra analysis indicated there were still small parts of second phase existing in 0.01 at % Ce^3+^ doped sample, which revealed that spectroscopy methods possessed better sensitiveness in smaller scale and lower concentration detection than XRD. As the doping content increased to 0.02 at %, a mass of second phase arose, as the major consequence, the optical transparency, mechanical property decreased and the PL spectra red shifted by the formation of second phase. The fluorescence thermostability was affected severely, where the PL intensity of 0.02 at % Ce^3+^ doped sample decreased to 48%, as temperature increased from 323 to 523 K. While, the 0.005 and 0.01 at % Ce^3+^ doped samples kept 90 and 85% of the 323 K intensity at 523 K, respectively. In conclusion, the doping content in MgAlON:Ce^3+^ fluorescent transparent ceramics system was not more than 0.01 at % in this work, an excess doping could produce second phase, which would seriously deteriorate the performance.

## Figures and Tables

**Table 1:** The relative densities, Vickers hardness, and internal quantum efficiencies of 0.005, 0.01, 0.02 at % Ce^3+^ doped MgAlON fluorescent transparent ceramics.

Ce^3+^ Doping Content (%)	Relative Density (%)	Vickers Hardness (GPa)	Internal Quantum Efficiency (%)
0.005	99.5 ± 0.2	14.0 ± 0.1	35
0.01	99.2 ± 0.5	13.7 ± 0.3	42
0.02	99.2 ± 0.3	13.8 ± 0.7	34
